# The use of local structural similarity of distant homologues for crystallographic model building from a molecular-replacement solution

**DOI:** 10.1107/S2059798320000455

**Published:** 2020-02-28

**Authors:** Grzegorz Chojnowski, Koushik Choudhury, Philipp Heuser, Egor Sobolev, Joana Pereira, Umut Oezugurel, Victor S. Lamzin

**Affiliations:** a European Molecular Biology Laboratory, c/o DESY, Notkestrasse 85, 22607 Hamburg, Germany

**Keywords:** model building, sequence similarity, *ARP*/*wARP*, macromolecular crystallography, loop building

## Abstract

The performance of automated protein model building usually decreases with resolution, mainly owing to the lower information content of the experimental data. This calls for a more elaborate use of the available structural information about macromolecules. Here, a new method is presented that uses structural homologues to improve the quality of protein models automatically constructed using *ARP*/*wARP*. The method uses local structural similarity between deposited models and the model being built, and results in longer main-chain fragments that in turn can be more reliably docked to the protein sequence. The application of the homology-based model extension method to the example of a CFA synthase at 2.7 Å resolution resulted in a more complete model with almost all of the residues correctly built and docked to the sequence. The method was also evaluated on 1493 molecular-replacement solutions at a resolution of 4.0 Å and better that were submitted to the *ARP*/*wARP* web service for model building. A significant improvement in the completeness and sequence coverage of the built models has been observed.

## Abstract 2

A novel method for the enhancement of automated protein model building using polypeptide fragments of homologous structures is presented.

## 1. Introduction   

Model building is a key step in macromolecular crystallo­graphic structure determination. With the availability of X-ray diffraction data to a resolution of better than 3.0 Å and an initial map of reasonable quality, model building can often be accomplished using automated approaches. The automated tools not only accelerate the model building itself but, more importantly, can also help to avoid subjectivity throughout the density-map interpretation process. The performance of crystallographic model-building methods is reduced at lower resolution owing to the lower information content of the data (Karmali *et al.*, 2009 ▸). For these cases the protein backbone models become fragmented, may contain insertions, deletions or incorrect connections, and may become difficult to assign to the target sequence (Chojnowski *et al.*, 2019 ▸).

A common way to assist automated model building in low-resolution and/or noisy electron-density maps is to complement the data with available *a priori* geometrical information in the form of larger building blocks: structural fragments. Secondary-structural elements can be used for the initial interpretation of the maps with template matching in recip­rocal space (Terwilliger, 2003 ▸) and real space (Sheldrick, 2010 ▸), real-space pattern recognition (Langer *et al.*, 2008 ▸) or graph-based approaches (Chojnowski *et al.*, 2015 ▸). There are also approaches that can use tertiary-structure elements for initial map interpretation (Usón & Sheldrick, 2018 ▸). Partial models can be expanded using databases of short continuous polypeptide fragments (Terwilliger, 2004 ▸; Chojnowski *et al.*, 2019 ▸). It has been shown that given the approximate coordinates of the C^α^ atoms of a protein, a full main chain can be built using short continuous fragments derived from a relatively small database of other proteins (Jones & Thirup, 1986 ▸). A related approach was used to correct for insertions and deletions, and to reduce the fragmentation of automatically traced main-chain models by building loops (Cowtan, 2012 ▸). As these approaches use short continuous fragments, typically up to six residues in length (Jones *et al.*, 1991 ▸; Cowtan, 2012 ▸), they can be used to fix local deficiencies in the model. To build longer loops, a selection of fragments from structures similar to the target is required (Cowtan, 2012 ▸).

Structures similar to a target protein, which can potentially provide longer fragments for model building, can be identified using sequence-alignment tools. It is well known that sequence and structure similarities are mutually interrelated and that high sequence similarity almost always implies similarity in the corresponding 3D structures (Krissinel & Henrick, 2004 ▸). The opposite, however, is not necessarily true and a lack of sequence similarity does not necessarily mean that the 3D structures are dissimilar (Söding, 2005 ▸). As a rule of thumb, one expects that a model with greater than 35% sequence identity will be structurally close enough to the target to enable a successful molecular-replacement (MR) solution (Abergel, 2013 ▸). There are, however, known examples of very similar protein structures with much lower sequence identity (Krissinel & Henrick, 2004 ▸). In contrast to MR applications, where overall fold similarity is required, model building can benefit from the availability of structures with local similarity to the target on a domain or even structural motif level, which can occur in proteins with low sequence similarity (Alva *et al.*, 2015 ▸).

Structural homologues with high sequence identity to the target protein can assist in the completion of large parts of a model that has already been refined and its fragments assigned to the sequence. A recent study (van Beusekom, Joosten *et al.*, 2018 ▸) showed that in over 24 000 protein models deposited in the PDB (Berman *et al.*, 2000 ▸), absent parts of the structure of up to 30 residues in length could be automatically identified and built into the electron density using the available structures of homologous proteins. The identification was based on sequence alignment of structural homologues with a relatively high (75% or more) sequence identity to the target.

The use of homologous structures for the completion of initial models, however, is more complex. During the process of automated model building the structure is typically evolving in an iterative manner. Throughout the evolution, the built model consists of several fragments with gaps between them. A sequence alignment with homologous protein structures may assist fragment extension and accelerate model building, although the backbone fragments may not always be reliably assigned to the sequence, particularly in models built at a lower resolution. In such cases, sequence alignment of a partial model with homologues may not be straightforward.

Here, we present a new method for the automated extension of fragmented crystallographic protein models before they are docked to the sequence. This method, referred to as ‘homology-based model extension’, is based on the use of local structural similarity between polypeptide stretches of homologous proteins (hereafter referred to as fragments), including cases in which the sequence similarity between the protein structures is low.

## 2. Materials and methods   

### 2.1. Overview of the homology-based model-extension method   

Model building using *ARP*/*wARP* proceeds in an iterative manner (Langer *et al.*, 2008 ▸) and one intermediate model is produced at each iteration. Homology-based model extension was developed for application to such an intermediate and it requires a backbone-only protein model and the target protein sequence. The following steps are then undertaken.

(i) For each chain in the target sequence, homologous protein structures are identified and downloaded from the PDB (Section 2.2).
(ii) For each continuous backbone fragment of longer than ten residues (a ‘query fragment’), structurally similar fragments from the homologous structures are identified and are accepted if they match with an r.m.s.d. below a certain threshold (Section 2.3). The threshold was defined using a training set of structures (Sections 2.5, 2.6 and 2.7).
(iii) This ‘structural alignment’ is carried out for all query fragments and their matching fragments (Section 2.4).
(iv) The query and the aligned matching fragments are successively merged and the main chain is constructed (Section 2.8).
(v) Finally, the merged model is docked to the target sequence and model building is iterated following the *ARP*/*wARP* protocol.

The developed method was evaluated using three test sets prepared as described in Section 2.9. The size of the target structure in the asymmetric unit was estimated using an empirical formula, as described in Section 2.10.

### 2.2. Selection of homologous protein structures from the PDB   

For each chain in the target sequence, up to 50 homologous protein structures are identified based on sequence similarity using *PHMMER* version 3.1b1 (Eddy, 2011 ▸). The sequences of the PDB structures are selected from the *MrBUMP* (Keegan & Winn, 2007 ▸) files within the *CCP*4 package (Winn *et al.*, 2011 ▸) based on the *E*-value for the sequence bit score of the best single domain, as defined in the *PHMMER* manual. In essence, we search for structures sharing at least one evolutionarily conserved domain region with the target sequence. Only structures with an *E*-value of below 10^−5^ (the number of expected hits in a database of this size containing only random sequences) are downloaded from the PDBe archive (Velankar & Kleywegt, 2011 ▸), which requires network access.

### 2.3. Structural similarity search and alignment   

The structures selected using *PHMMER* were used in a search for structural similarity of the query fragments using *GESAMT* (version 1.15; Krissinel, 2012 ▸) as included in the *CCP*4 suite version 7.0.065, without taking any sequence similarity into account. For computational reasons the search is carried out in two steps. Firstly, *GESAMT* is used to prepare a shortlist of structures for each query fragment. During the second step *GESAMT* is run on a shortlist of fragment–structure pairs and for each of them provides a matching fragment to the query fragment, an r.m.s.d. of the match and the transformation matrices. The fragments from the homologues were accepted as ‘matching fragments’ if they superimpose on the query fragments with an r.m.s.d. below a certain threshold. The determination of the optimum threshold is described below.

In its default configuration* GESAMT* does not align query fragments with much larger structures. Therefore, two specific instructions for *GESAMT* were used for the alignment of structures of any size and the identification of matches for at least 80% of the query-fragment length (-min1=0.8 and -min2=0.0).

### 2.4. Structural alignment   

A matching fragment from the structural similarity search is superimposed on the C^α^ atoms of the corresponding query fragment. The same transformation is used to place the flanks (ten residues before and after the matching fragment). The matching fragments from the homologous structures superimposed on the query fragments together with their flanking residues are called ‘aligned matching fragments’.

### 2.5. Preparation of a set of fragments for training   

The training fragments were prepared to mimic the fragments from an intermediate *ARP*/*wARP* model in order to develop the method and to derive important parameters. 1000 protein crystal structures were taken from the PDB as of 21 October 2018. The structures were selected at random, ignoring any potential structural or sequence similarity between them, provided that they contained at least one continuous chain with 70 (or more) residues and were determined at a resolution of 3.0 Å or better. Parts of the structures modelled in multiple conformations were excluded. From each structure, nine continuous test fragments of lengths of 10, 15, 20, 25, 30, 35, 40, 45 and 50 residues were extracted. Each training fragment was taken from a random location, but under the condition that there were continuous flanks of ten residues preceding the fragment and ten residues following the fragment.

It has been observed that crystal structures of the same or similar proteins, which are refined independently against their X-ray data, superimpose with an r.m.s.d. value dependent on the resolution of the X-ray data (Cruickshank, 1999 ▸; Carugo, 2003 ▸). A fragment from an intermediate, not finally refined, *ARP*/*wARP* model is expected to have a higher r.m.s.d. to its homologous structure in the PDB. Therefore, to make the training fragments more realistically imitate an *ARP*/*wARP* intermediate model, they were subjected to a uniform random coordinate error with an overall r.m.s.d. of 0.5 Å; protein stereochemistry was ignored. The r.m.s.d. value of 0.5 Å was estimated to be a good approximation of the main-chain coordinate error based on intermediate *ARP*/*wARP* protein models built at resolutions between 2.0 and 3.0 Å in a previous study (Chojnowski *et al.*, 2019 ▸). From 1000 protein structures, a set of 9000 short backbone fragments with an introduced coordinate error for training the parameters of the method was obtained.

The obtained training fragments were regarded as mimicking the query fragments. For each training fragment a subset of PDB structures was identified using the sequence of the associated structure, as described in Section 2.2. The homologous matching fragments were identified in the relevant PDB subset and were structurally aligned with the query fragments using *GESAMT* as described in Section 2.3.

### 2.6. Differentiating ‘positive’ aligned structural fragments from ‘negative’ ones   

For the development of the method, we needed to evaluate how well the aligned matching fragments agree with the final structure (as deposited in the PDB) associated with the query fragments in the training set. For each structural alignment we counted the number of C^α^ atoms in the aligned matching fragments that were within a distance of 1.0 Å of a C^α^ atom in the final structure (hereafter called ‘matching C^α^ atoms’). Structural alignments with a number of matching C^α^ atoms higher than an arbitrarily selected threshold of 80% of the length of the matching fragment (together with its flanks) were marked as ‘positives’ and the remaining alignments as ‘negatives’. For each structural alignment the r.m.s.d. value between the query and the matching fragments, as reported by *GESAMT*, was stored.

### 2.7. Determination of the optimum r.m.s.d. threshold for the identification of matching fragments   

For most query fragments, several matching fragments can be found from different structures in the PDB. The r.m.s.d. values between the aligned C^α^ atoms can be calculated, but the probability that a matched fragment is locally similar to the query fragment remains to be estimated. We determined the maximum r.m.s.d. threshold for a match to be accepted so that the probability that the matched fragments are locally similar is maximized.

**Figure 1:**
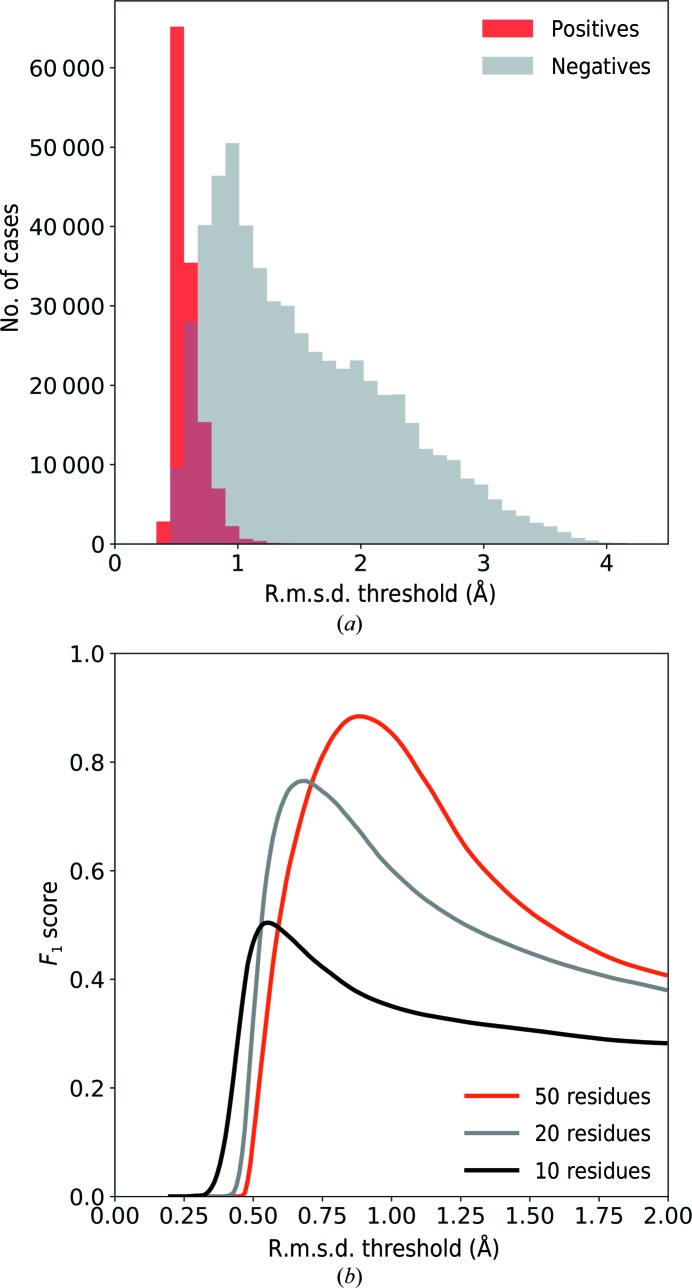
The r.m.s.d. thresholds for selecting matched fragments. (*a*) Distribution of the r.m.s.d. for fragments of 20 residues in length. Alignments with local structural similarity (‘positives’) and those without (‘negatives’) are indicated. (*b*) The *F*_1_ score as a function of the r.m.s.d. threshold for fragments of different lengths.

Each fragment in the training set (Section 2.5) was regarded as a test query fragment and was structurally compared with a subset of PDB structures with a best single-domain *E*-value below 10^−5^ (as described in Sections 2.2 and 2.4). The majority of fragment pairs were aligned with an r.m.s.d. below 1.0 Å (shown as ‘positives’ in Fig. 1 ▸ *a*). To estimate an r.m.s.d. threshold for the optimum selection of ‘positives’ and rejection of ‘negatives’, we used the *F*_1_ score as a similarity measure (Chinchor, 1992 ▸). The *F*_1_ score is a harmonic mean of recall (the fraction of the selected ‘positives’) and precision (the fraction of the ‘positives’ in the selection) of a binary classifier, and can be expressed as
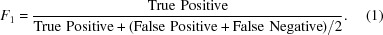
Fig. 1 ▸(*b*) shows the dependence of the *F*_1_ score on the r.m.s.d. threshold below which the matched fragments are accepted. The maxima of the *F*_1_ score correspond to the optimum r.m.s.d. thresholds, which are different for different fragment lengths (Table 1 ▸). The optimum r.m.s.d. threshold for a matched fragment to be accepted is about 0.5 Å for fragments of ten residues in length; for longer fragments it approaches a value of 1.0 Å, which was used as a limit to define ‘positives’. For practical implementation we used an r.m.s.d. threshold of 0.9 Å for fragments of 50 or more residues in length.

We found that a flank length of ten residues provides good results, but we have not systematically studied the impact of the flank length on the performance of the presented method. We note, however, that the flank length may significantly affect the number of accepted fragments (positives). For example, 30% fewer fragments of length 20 were accepted after increasing the flank length from ten to 20 residues.

### 2.8. Assembly of the fragments into the main chain   

To assemble a most likely backbone from overlapping query fragments and aligned matching fragments, we followed a consensus approach (Lundström *et al.*, 2001 ▸). Specifically, a backbone model is built based on the most common parts of the fragments and their fit to the electron-density map.

We start by encoding all fragments as a directed, weighted graph. Graph nodes correspond to the C^α^ atoms and are annotated with the *xyz* coordinates of their positions. Graph edges link successive C^α^ atoms in the direction of the fragments. Additionally, the edges are annotated with weights corresponding to the *ARP*/*wARP* score reflecting the fit of the main-chain atoms to the density, as described in Lamzin & Wilson (1997 ▸).

The obtained graph consists of many components, each corresponding to either a query or a structurally aligned matching fragment. The nodes and edges of the graph components are merged in three steps.

**Figure 2:**
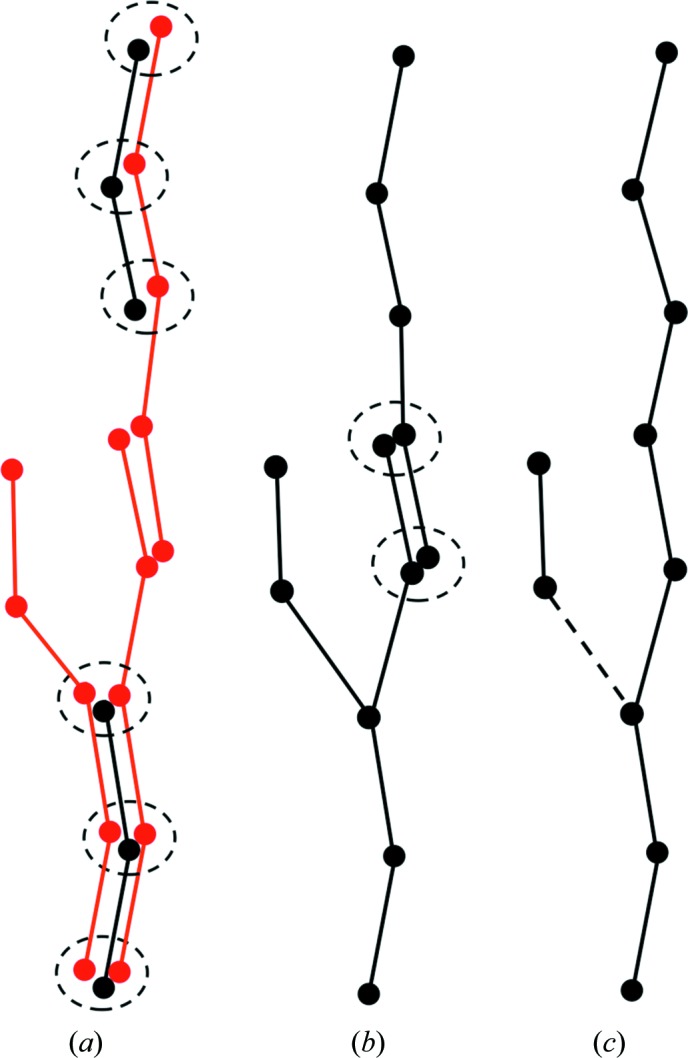
Schematic representation of the fragment-assembly algorithm. The graphs represent query fragments and aligned matching fragments (black and red, respectively; edge weights and directions are not shown for clarity). In the first step, graph nodes corresponding to the query fragments are merged with all remaining nodes within a distance of 1.0 Å (*a*). Next, the remaining nodes are merged with their neighbours within a distance of 1.0 Å in an arbitrary order (*b*). Finally, branching edges are removed (dashed line) (*c*).

*Step 1*. Each node corresponding to a query fragment from an intermediate *ARP*/*wARP* model is merged with all other nodes that are located within a distance of 1.0 Å. The resulting node has the average *xyz* coordinates of the merged nodes and also inherits their edges. As the distance between the successive C^α^ atoms in a *trans*-peptide is 3.8 Å, spheres with 1.0 Å radius centred at successive C^α^ atoms do not overlap with each other. Therefore, the nodes of the query fragments are merged independently in an arbitrary order (nodes within dashed circles in Fig. 2 ▸ *a*).
*Step 2*. After processing the query fragments, the remaining nodes from the aligned matching fragments are similarly merged. The order in which they are merged does not significantly affect the results; thus, the nodes are also processed in an arbitrary order (Fig. 2 ▸ *b*).
*Step 3*. When all nodes have been merged, redundant edges connecting the same nodes are also merged and their weights are summed. Cyclic paths, if present, are opened by removal of the edge with the lowest weight.

After the merging of nodes and edges, the graph may still contain branches of incoming or outgoing edges. These are resolved in an arbitrary order using local pruning. For each outgoing edge of a branched node, a set of paths, with each path being up to three edges in length, is identified using a depth-first search. For each path, a sum of the weights of its edges is computed. The maximum sum is assigned to the outgoing edge in question. When all outgoing edges are processed, the edge with the highest maximum sum is kept and the others are removed (dashed edge in Fig. 2 ▸ *c*). Branched incoming edges are similarly resolved.

Possible overlaps between different paths that contain common nodes and are running in opposite directions are resolved iteratively. The path with the higher sum of edge weights is retained and the edges connecting common nodes in the second path are removed from the graph. A full-atom model for the selected paths with defined chain direction and containing C^α^-atom candidates is built using the *ARP*/*wARP* main-chain tracing algorithm (Morris *et al.*, 2002 ▸).

### 2.9. Preparation of the two test sets and their annotation with *R*_free_ values   

From June 2017 to February 2019, 12 823 model-building tasks were submitted to the *ARP*/*wARP* web service (https://arpwarp.embl-hamburg.de/). The majority of these (88%) were protein model-building tasks starting from an existing model. To eliminate redundant cases, we clustered them using *CD-HIT* (Li & Godzik, 2006 ▸) at a 95% sequence-identity level, which resulted in test set I containing structures with 1753 unique sequences. We then aligned each unique sequence with the sequences of crystal structures deposited in the PDB before June 2017 using *Protein BLAST* version 2.2.26+ (Altschul *et al.*, 1997 ▸) and noted the corresponding sequence identity.

Of these 12 823 model-building tasks, 4242 were submitted to the *ARP*/*wARP* web service from the *CCP*4 online (Krissinel *et al.*, 2018 ▸) MR pipelines *MrBUMP* (Keegan & Winn, 2007 ▸), *MORDA* (Vagin & Lebedev, 2015 ▸) and *BALBES* (Long *et al.*, 2008 ▸). Of these, 2164 tasks had X-ray data extending to a high-resolution limit of between 2.0 and 3.0 Å, 1095 to better than 2.0 Å and 983 to worse than 3.0 Å. The MR solutions were subjected to ten cycles of restrained refinement using *REFMAC* (Murshudov *et al.*, 2011 ▸) and the *R*_free_ factor obtained for 5% of the reflections is hereafter defined as the ‘initial *R*_free_’. These model-building tasks were also clustered at 95% sequence identity using *CD-HIT*. From each sequence cluster the model-building task with the lowest initial *R*_free_ was selected. This resulted in test set II containing 811 tasks with the high-resolution limit within the 2.0–3.0 Å range, 444 tasks at a resolution better than 2.0 Å and 238 tasks with a high-resolution limit between 3.0 and 4.0 Å. Tasks with a data resolution of worse than 4.0 Å, which constituted 3% of the total number of cases, were not used.

The structures in test set II were rebuilt using *ARP*/*wARP* version 8.0 with default parameters and with the use of the homology-extension module presented in this work. To minimize model bias, the homologues for the model extension were selected from structures deposited in the PDB before June 2017, *i.e.* before the tasks comprising the test set were submitted to the *ARP*/*wARP* web service. Special care was taken to obtain reliable estimates of the *R* values for the final models. The central concept of model building with *ARP*/*wARP* is the use of ‘free atoms’ for the sparse representation of electron-density maps. The free atoms are removed from the final models only if their sequence coverage exceeds 80% (Morris *et al.*, 2004 ▸). Otherwise, they are kept in order to preserve the representation of the electron density where the protein model is not built, which may affect the final *R* values. To avoid this issue, all of the final models were subjected to *ARP*/*wARP* solvent modelling (Lamzin & Wilson, 1993 ▸) after removing all free atoms. The resulting *R*_free_ values, hereafter defined as ‘final *R*_free_’ values, were used in further analysis.

### 2.10. Estimation of the size of the model to be built   

The sequence length for a homomer or heteromer was derived from the input sequence file. The total number of residues expected in the asymmetric unit was derived from the product of the sequence length and the number of sequence copies if both were given in the sequence file. Otherwise, the number of sequence copies was derived from the estimated solvent content and the sequence length computed using the following empirical formulas implemented in *ARP*/*wARP* 8.0,
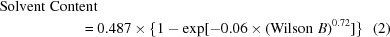


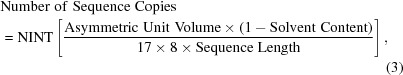
where Wilson *B* is the Wilson plot *B* factor (Å^2^), NINT is the nearest integer, 17 is the volume (in Å^3^) occupied by an ‘average’ non-H atom in a protein structure and 8 is the average number of atoms in a protein residue. The formulas (2) and (3) are routinely used in *ARP*/*wARP* for estimating the expected number of residues in the asymmetric unit. They were introduced in 2008 and were derived based on over 30 000 experimental diffraction data sets available in the PDB at the time. The Wilson plot *B* factor is estimated following Popov & Bourenkov (2003 ▸).

The completeness of an MR model is defined as the ratio of the number of residues that it contains to the total number of expected residues in the asymmetric unit.

### 2.11. Model-quality metrics   

Reference models that could be used for model validation are not available for all test-set structures. Therefore, we used model-validation metrics based on the total number of residues in the final models. Model ‘completeness’ is the ratio of the total number of residues in a final model to the expected number of residues in the asymmetric unit (see Section 2.10 for details). ‘Sequence coverage’ is the ratio of the number of residues assigned to the target sequence to the expected number of residues in the asymmetric unit.

### 2.12. Implementation   

The benchmarks were performed using the *GNU Parallel* software (Tange, 2015 ▸). The developed method has been implemented with the use of the *CCP*4 (Winn *et al.*, 2011 ▸) and *cctbx* (Grosse-Kunstleve *et al.*, 2002 ▸) utilities and libraries. The method has been provided in the current web service version of *ARP*/*wARP* 8.0 and will be made available to the community within the next *ARP*/*wARP* software release.

## 3. Results   

### 3.1. Homologues that are already available in the PDB   

We have attempted to estimate the number of model-building tasks that have recently been submitted to the *ARP*/*wARP* web service and already had a homologous protein model available in the PDB before the model-building task was launched.

**Figure 3:**
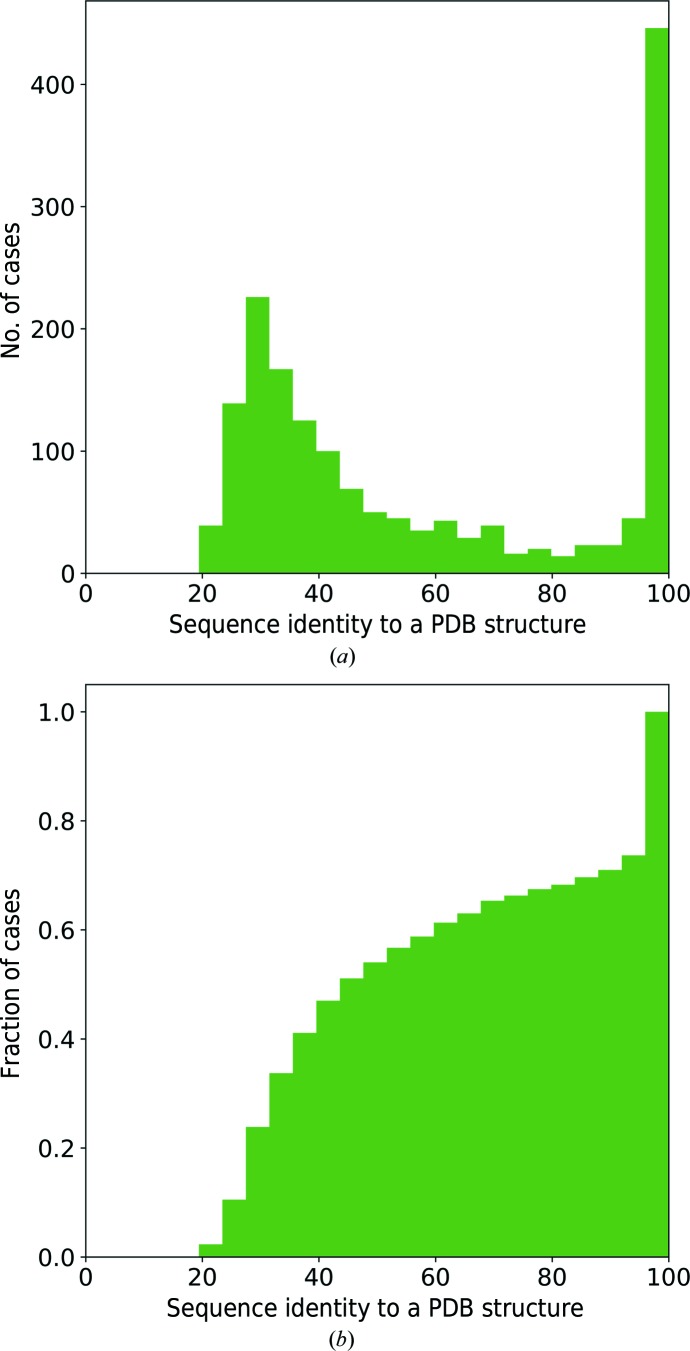
(*a*) Distribution of the highest sequence-identity match of each unique sequence of the *ARP*/*wARP* web service model-building tasks (June 2017 to February 2019) to the protein structures already available in the PDB; (*b*) the corresponding cumulative distribution.

For each unique sequence in test set I (Section 2.9), the highest sequence-identity match to the structures in the PDB is shown in Fig. 3 ▸. We observed that for 29% of the unique sequences (corresponding to 32% of the model-building tasks) a close homologue with greater than 90% sequence identity was already available in the PDB before the commencement of the model-building task. Furthermore, for 65% of the unique sequences (59% of the model-building tasks) a closest available homologue had 35% (or higher) sequence identity: the value attributed to a highly probable structural similarity (Krissinel, 2007 ▸).

### 3.2. Evaluating the method on the test sets   

To evaluate the potential benefit of the new homology-extension method, we compared model-building performance with *ARP*/*wARP* 8.0 for the MR tasks submitted to the *ARP*/*wARP* web service (defined as test set II in Section 2.9). We observed that the use of homology-based fragment extension generally improves the quality of built models at various levels of model completeness.

**Figure 4:**
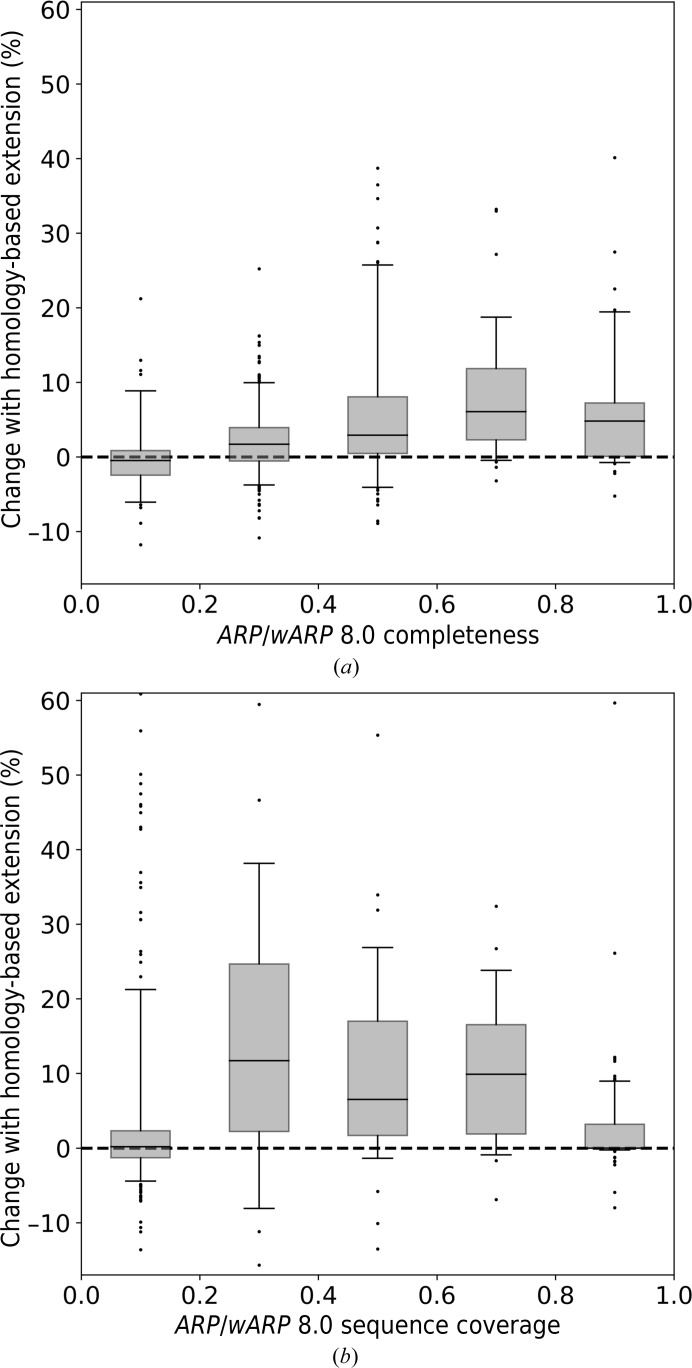
Improvement in model building for test set II at resolutions between 2.0 and 3.0 Å. (*a*) The fraction of residues built; (*b*) the sequence coverage. Box-plot whiskers correspond to the 5th and 95th percentiles.

**Figure 5:**
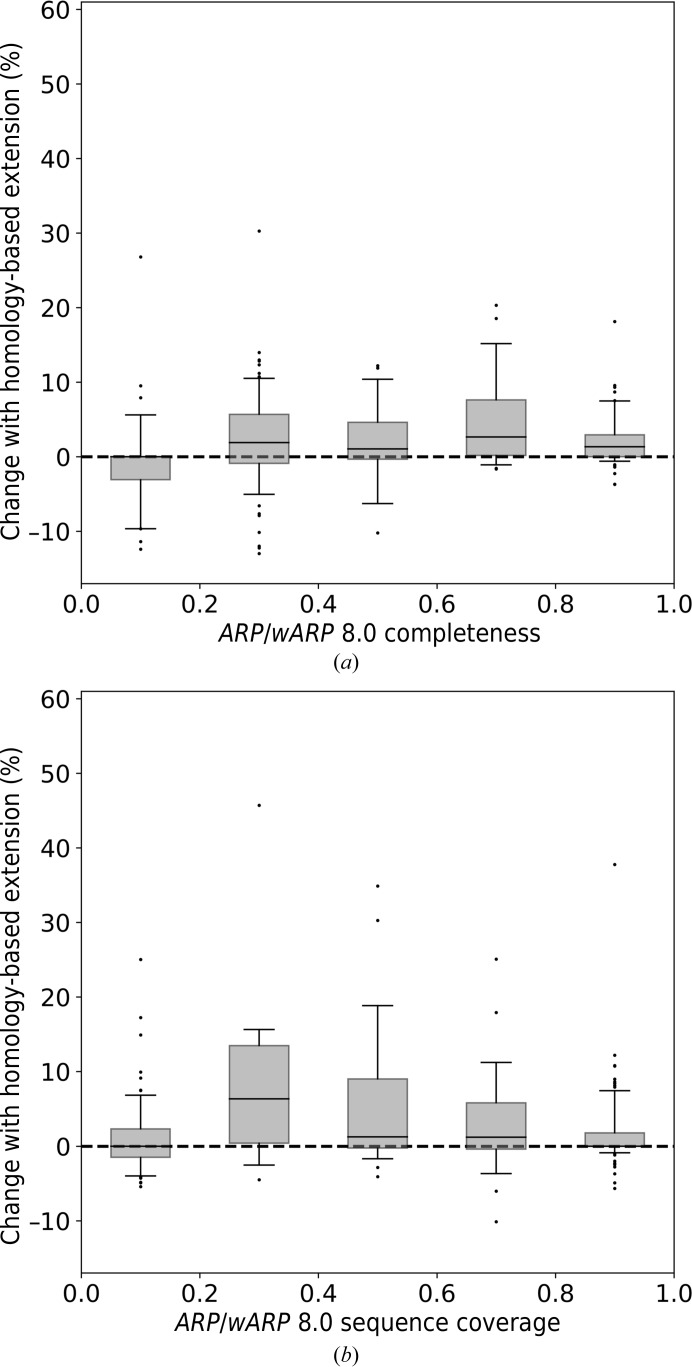
Improvement in model building for test set II at resolutions better than 2.0 Å. (*a*) The fraction of residues built; (*b*) the sequence coverage. Box-plot whiskers correspond to the 5th and 95th percentiles.

With X-ray data extending to a resolution of between 2.0 and 3.0 Å, the use of homology-based extension noticeably increases both the completeness and the sequence coverage of the built models (Fig. 4 ▸). The effect is pronounced for models built to a completeness of 50% or higher. Similarly, at a resolution worse than 3.0 Å the most significant improvement in model completeness is obtained for models that could be built to at least 50% complete with *ARP*/*wARP* 8.0 defaults (Supplementary Fig. S7). At a resolution better than 2.0 Å the improvement is also pronounced, although to a lesser extent, as most of the models can be built to a very high completeness anyway (Fig. 5 ▸).

At a resolution better than 3.0 Å the model improvement depends more on the quality and completeness of the initial MR solution (Supplementary Figs. S1 and S4) than on the resolution of the diffraction data set (Supplementary Figs. S3 and S6). By contrast, at a resolution worse than 3.0 Å an improvement can be obtained up to about 3.4 Å resolution, which seems to be the limit for automated model building with the current *ARP*/*wARP* implementation (Supplementary Figs. S8 and S10).

**Figure 6:**
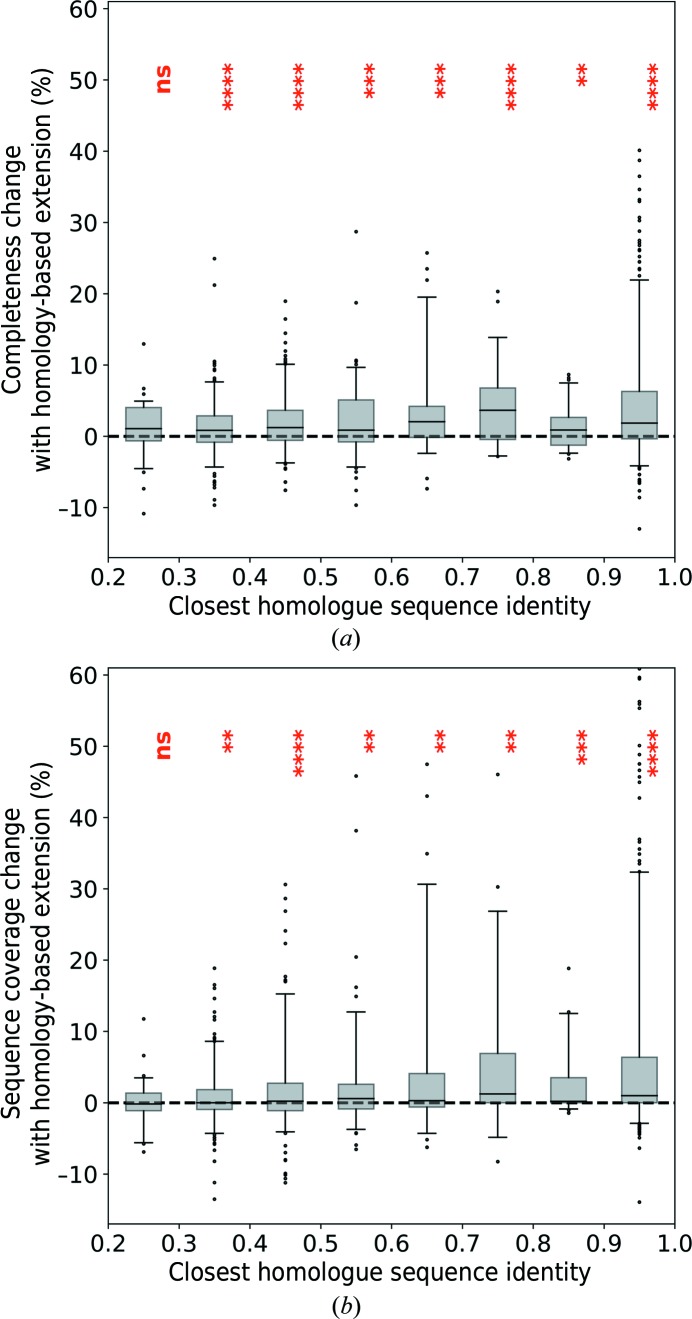
The improvement of model-building results in the complete test set II as a function of the sequence identity to the closest available homologue. (*a*) Relative change in model completeness, (*b*) relative change in sequence coverage. Box-plot whiskers correspond to the 5th and 95th percentiles. The significance level of a one-sided Student’s *t*-test for the average improvement is marked above the boxes (ns, nonsignificant; *p*-values below 0.05, 0.01, 0.001 and 0.0001 are denoted with one to four stars, respectively).

We note that the use of the homology-based extension module results in a statistically significant improvement in the model completeness and sequence coverage when the sequence identity to the closest homologue is 30% or higher (Fig. 6 ▸).

### 3.3. Elaboration on a model-building example at 2.7 Å resolution   

This example is a cyclopropane fatty acid phospholipid synthase (CFA synthase) from *Lactobacillus acidophilus*. The model was solved using *Phaser-MR* (McCoy *et al.*, 2007 ▸) and refined at 2.7 Å resolution (Ma *et al.*, 2019 ▸). The deposited model (PDB entryy 5z9o) contained 798 residues corresponding to two molecules of CFA synthase in the asymmetric unit and was refined to *R* and *R*_free_ factors of 17.5% and 21.3%, respectively.

**Figure 7:**
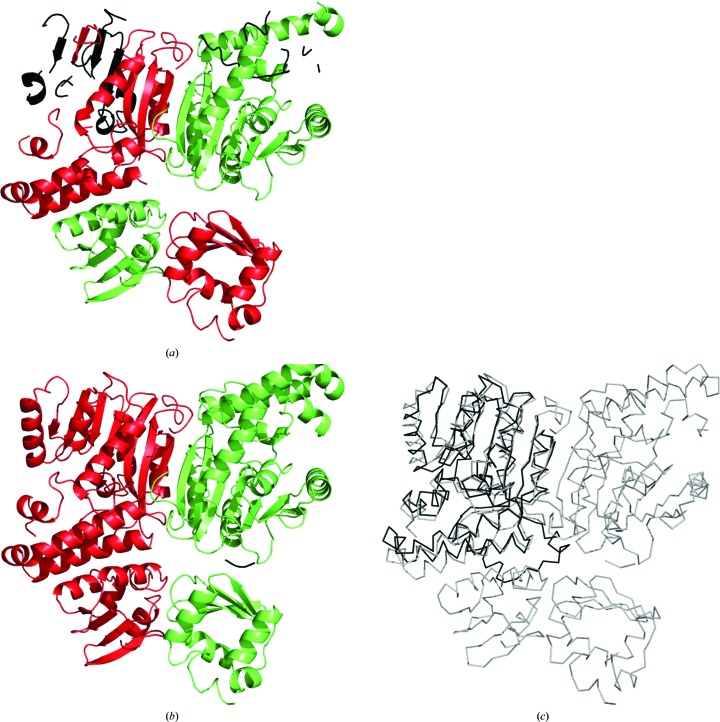
*ARP*/*wARP* models of CFA synthase built at 2.7 Å resolution using default parameters. Parts of the models that were not assigned to the sequence are presented in black, while other chains are shown in red and green. The models were built (*a*) without homology-based extension and (*b*) with homology-based extension. (*c*) The closest homologue and the MR search model (PDB entry 3hem), shown in black, superposed onto the *ARP*/*wARP* model from (*b*), shown in grey.

Structure solution was also attempted within the *MrBUMP* pipeline, where the MR solution was obtained using *MOLREP* (Vagin & Teplyakov, 2010 ▸), and the result was forwarded to the *ARP*/*wARP* web service for model building. The input model with 518 residues had initial *R* and *R*_free_ factors of 41% and 45%, respectively. 683 residues were built in 20 fragments, with 633 residues assigned to the sequence (Fig. 7 ▸ *a*). The addition of the homology-based extension module resulted in a better *ARP*/*wARP* model with 734 residues built in six fragments and almost all of them, 730 residues, assigned to the sequence (Fig. 7 ▸ *b*). The final crystallographic *R* and *R*_free_ factors for the *ARP*/*wARP* models with the built solvent were 23% and 30%, respectively, without and 18% and 24%, respectively, with the use of homology-based extension.

**Figure 8:**
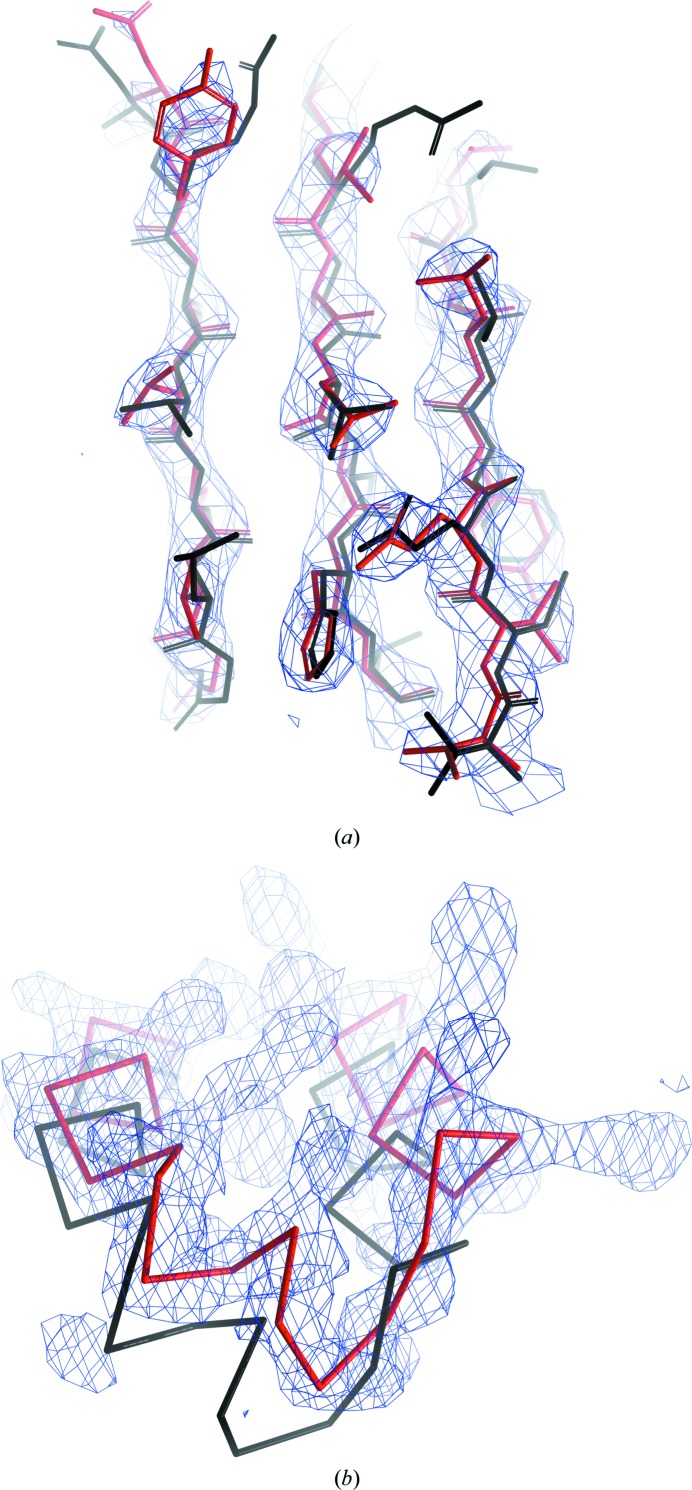
Close-up views of the *ARP*/*wARP* model of CFA synthase built at 2.7 Å resolution using default parameters (red) with the superposed closest homologue (PDB entry 3hem, black): (*a*) core region of the protein with low sequence variability and well conserved structure, (*b*) solvent-exposed part where sequence and structure diverge (side chains are not shown for clarity). The final 2*F*_o_ − *F*_c_ maps are contoured at the 1.5σ density level above the mean.

Although for homology-based extension we used only structures that were deposited in the PDB before the model-building task of CFA synthase was undertaken, a number of related lipid synthase structures were already available. These structures contain only the larger C-terminal domain of 280 residues and the closest available homologue, a cyclopropane mycolic acid synthase from *Mycobacterium tuberculosis* (PDB entry 3hem; D. Barkan, Z. Liu, J. C. Sacchettini & M. S. Glickman, unpublished work), has 37% sequence identity to the C-terminal domain of the target. In fact, the MR solution by *MrBUMP* used exactly the same structure. Structural superposition of the MR search model with the deposited CFA synthase model revealed that 247 C^α^ atoms out of 366 in a monomer superimposed with an r.m.s.d. of 1.7 Å, indicating a similarity in their fold (Fig. 7 ▸ *c*). However, the models differ substantially in many regions and only 31% of the pairs of C^α^ atoms are matched with a displacement of less than 1.0 Å (Fig. 8 ▸). We observed a similar situation for the second monomer of the model.

### 3.4. The relationship between the quality of an MR solution and the performance of the model-building procedure   

**Figure 9:**
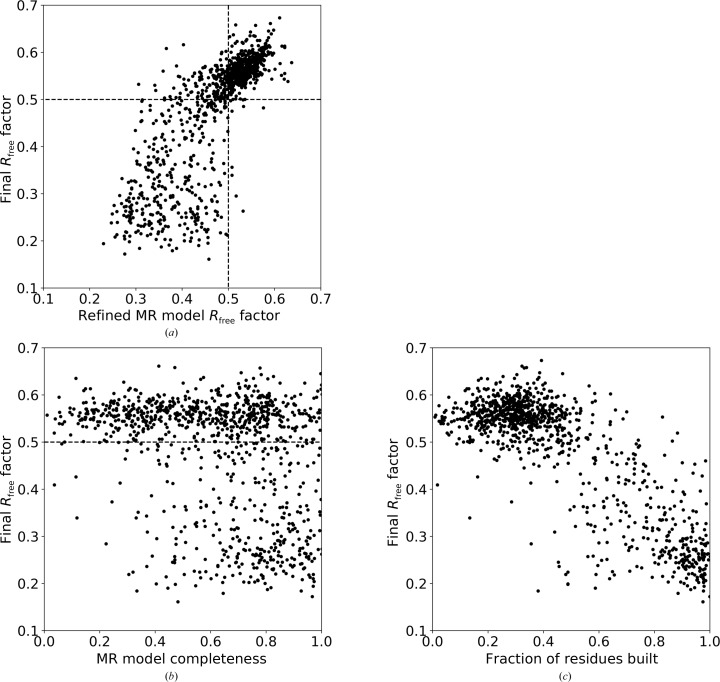
The comparison of the final *R*_free_ for the models built by *ARP*/*wARP* with homology-based extension (without free atoms and following *ARP*/*wARP* solvent building) as a function of (*a*) the *R*_free_ value for the initial MR solution, (*b*) the completeness of the MR model and (*c*) the fraction of the residues built.

To investigate the success rate of model building for MR solutions, we have additionally evaluated the dependence of *R*_free_ values for the initial MR solution and for the built *ARP*/*wARP* model (Fig. 9 ▸ *a*; see Section 2.9 for the definition of *R*_free_ for the initial and the built models). Model building for MR solutions with an *R*_free_ below 50% can frequently be accomplished automatically, particularly if the X-ray data extend to a resolution better than 2.0 Å (Fig. 9 ▸ *a*).

Clearly, high initial *R*_free_ values may reflect the quality of the MR solution: the accuracy of model placement, the level of model completeness and its similarity to the target. We attempted, however, to investigate whether an estimate of the model completeness (see Section 2.10) could be correlated with the final model *R*_free_ value. Indeed, we observed that for MR solutions that are less than 50% complete only a very few models could be automatically built (Fig. 9 ▸ *b*).

We note that the use of *R*_free_ was proposed for cross-validation in order to avoid overfitting when refining a model containing incorrectly built regions against the diffraction data despite stereochemical restraints (Brünger, 1992 ▸). Similarly, *R*_free_ may be used for validation of a model-building procedure, as more complete *ARP*/*wARP* models generally have lower final *R*_free_ values (Fig. 9 ▸ *c*).

**Figure 10:**
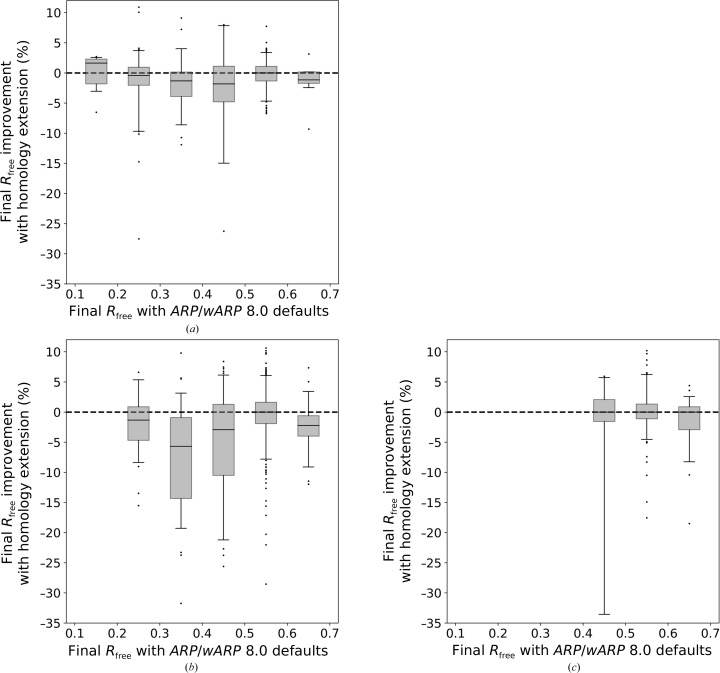
The influence of the homology-based extension on the *R*_free_ value for models built with *ARP*/*wARP* version 8.0 at resolutions (*a*) better than 2.0 Å, (*b*) between 2.0 and 3.0 Å and (*c*) below 3.0 Å. Box-plot whiskers correspond to the 5th and 95th percentiles.

For a demonstration of the overall validity of the *ARP*/*wARP* procedure (with and without homology-based extension) *R*_free_ was useful. Homology-based extension readily reduces final model *R*_free_ factors for *ARP*/*wARP*-built models with a final *R*_free_ between 25% and 50%. In other words, apart from a few prominent cases, the new method improves the quality of the models that can be at least partially built with the default settings. The effect is most pronounced for the resolution range between 2.0 and 3.0 Å (Fig. 10 ▸ *a*), but is also seen at resolutions better than 2.0 Å (Fig. 10 ▸ *b*) and worse than 3.0 Å (Fig. 10 ▸ *c*).

At the same time, the results presented in this section should not be misinterpreted as a recommendation to always use *R*_free_ to monitor model building. Indeed, setting aside a fraction of the X-ray data for cross-validation reduces the amount of available data and thus may adversely affect the performance of automatic model building with *ARP*/*wARP*, particularly at lower resolution. We observed that the use of *R*_free_ led to a slight increase in the number of built and docked residues in less than 40% of cases, while in the remaining cases the number of built and docked residues was reduced. Overall, a somewhat higher fraction of residues were built and docked when the use of *R*_free_ was turned off (Table 2 ▸). These results agree with the earlier observation that the exclusion of even as few as 5% of the free reflections from the diffraction data may noticeably increase the noise level in maps (Urzhumtsev *et al.*, 2014 ▸) and apparently also affect their interpretability.

## 4. Discussion and conclusions   

With over 130 000 crystal structures currently available in the PDB, it may be possible to find a homologue for many newly crystallized proteins. Indeed, the MR method accounts for almost 80% of the solved structures deposited in the PDB. Apart from assisting in structure solution using MR, homology has been exploited for crystallographic model building and refinement when a sequence assignment is available (van Beusekom, Joosten *et al.*, 2018 ▸; van Beusekom, Touw *et al.*, 2018 ▸; Kovalevskiy *et al.*, 2016 ▸; Nicholls *et al.*, 2012 ▸; Schröder *et al.*, 2010 ▸; Smart *et al.*, 2012 ▸; Headd *et al.*, 2012 ▸). It is intriguing that the majority of model-building tasks that have recently been submitted to the *ARP*/*wARP* web service had a homologous structure with a sequence identity of 35% (or greater) already available in the PDB.

We demonstrate that homology-based fragment extension with *ARP*/*wARP* improves the completeness and sequence coverage for many models that could otherwise be built to a lower extent. Structures with data to a resolution within the 2.0 to 3.0 Å resolution range may benefit most, as their experimental data have a lower information content.

For a deeper insight into the performance of homology-based extension, we analysed a 2.7 Å resolution MR model of a CFA synthase submitted to the *ARP*/*wARP* web service for model building. The input MR model was 58% complete, with an initial *R*_free_ of 45%. The structure could be built to 86% completeness with *ARP*/*wARP* 8.0, although in many fragments. The use of homology-based extension provided a more complete model with almost all of the residues docked to the sequence. In particular, all of the residues in the substrate-binding site were only correctly built in the model built using homology-based extension. We note that the efficient exploitation of local, rather than overall, structural homology plays the key role in the presented method.

In the presented work, we used a relatively simplistic approach for selecting homologous structures based on sequence alignment. The use of more sophisticated approaches for the detection of local structural similarity (see, for example, Alva *et al.*, 2016 ▸; Hildebrand *et al.*, 2009 ▸) could be considered in future research. Similarly, we consider that the optimization of other parameters and hyperparameters of the presented method (for example, flank lengths and r.m.s.d. threshold selection criteria) may be the subject of future research. In this work, only 648 MR solutions from test set II were good enough (that is, with an MR solution *R*_free_ below 50%) to initiate successful automated model building using the current *ARP*/*wARP* implementation. A systematic and rigorous optimization of the parameters of the method would require splitting the test set into smaller training, test and validation sets, which would reduce the reliability of the presented results and introduce the threat of overfitting.

The presented methods were evaluated using structures solved using MR. However, apart from the availability of homologues, no limitations are expected for the application of the developed methodology to structures solved using other approaches, for example experimental phasing (McCoy & Read, 2010 ▸) or fragment-based MR (Sammito *et al.*, 2014 ▸; Jenkins, 2018 ▸).

## Supplementary Material

Supplementary Figures. DOI: 10.1107/S2059798320000455/ip5005sup1.pdf

## Figures and Tables

**Table 1:** Optimum r.m.s.d. thresholds and corresponding *F*_1_ score values used for the determination of matching fragments

Fragment length (residues)	R.m.s.d. threshold (Å)	*F*_1_ score
10	0.55	0.50
15	0.62	0.68
20	0.69	0.77
25	0.75	0.82
30	0.79	0.83
35	0.82	0.84
40	0.85	0.88
45	0.86	0.89
50	0.88	0.89

**Table 2:** *ARP*/*wARP* model-building performance for MR cases with models with an initial *R*_free_ below 50%

		5% of X-ray data set aside for *R*_free_		All X-ray data used for model building and refinement	
Resolution of the X-ray data (Å)	No. of model-building cases	Average fraction of residues built (%)	Average sequence coverage (%)	Average fraction of residues built (%)	Average sequence coverage (%)
2.0–3.0	242	69.7	55.0	70.3	56.2
2.0 or better	196	82.2	73.9	82.5	75.2
