# Supplementary material for: The use of local structural similarity of distant homologues for crystallographic model building from a molecular-replacement solution
Source: Acta Crystallogr D Struct Biol. 2020 Feb 28;76(Pt 3):248–60. doi: 10.1107/S2059798320000455 (PMC7057216; doi:10.1107/S2059798320000455)
Supplement: Supplementary file 1 [file d-76-00248-sup1.pdf]

STRUCTURAL
BIOLOGY

Volume 76 (2020)

Supporting information for article:

The use of local structural similarity of distant homologues for crystallographic model building from a molecular-replacement solution

Grzegorz Chojnowski, Koushik Choudhury, Philipp Heuser, Egor Sobolev, Joana Pereira, Umut Oezugurel and Victor S. Lamzin

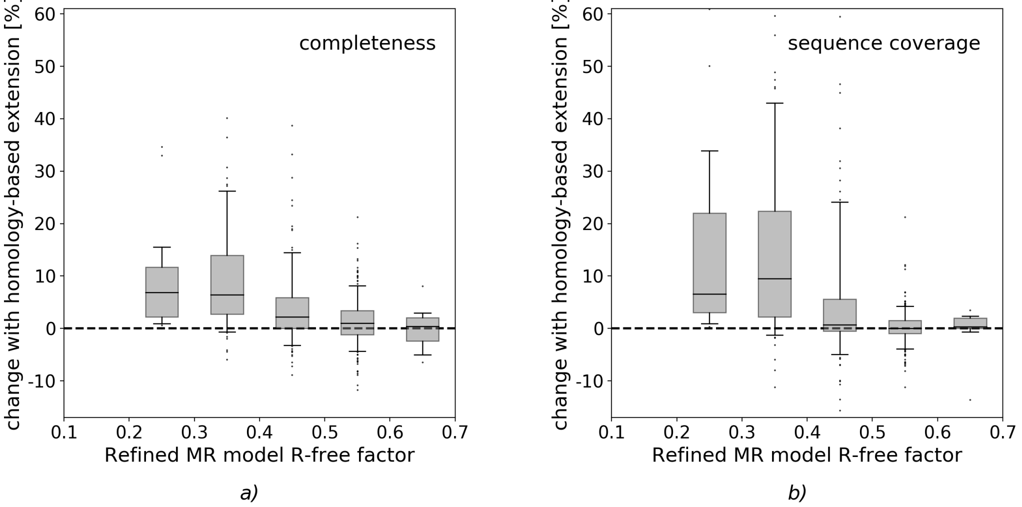

Figure S1 The improvement in model building for test set II at a resolution between 2 and 3 Å as a function of refined MR model R-free factor. a) the fraction of residues built; b) sequence coverage. Box plot whiskers correspond to the 5th and 95th percentile.

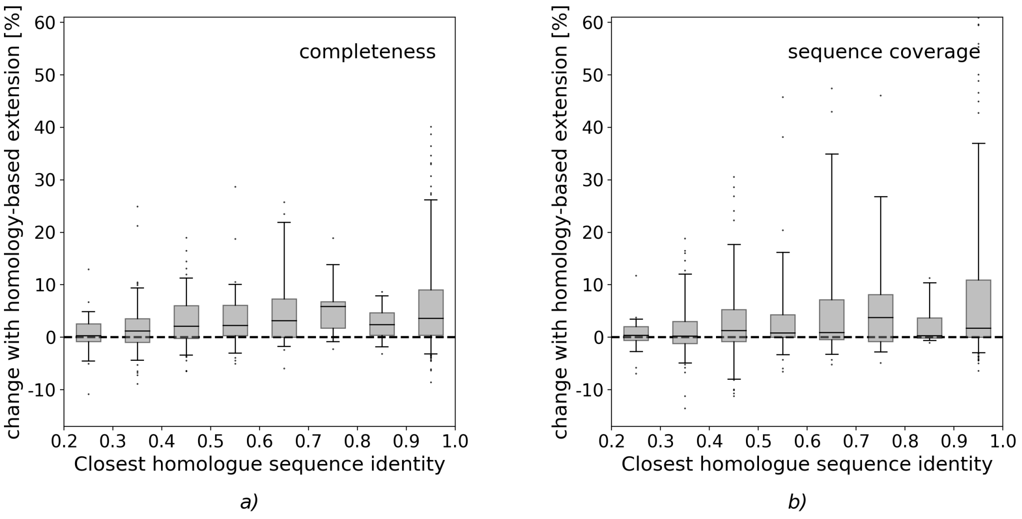

Figure S2 The improvement in model building for test set II at a resolution between 2 and 3 Å as a function of sequence identity to a closest homologue used for model extension. a) the fraction of residues built; b) sequence coverage. Box plot whiskers correspond to the 5th and 95th percentile.

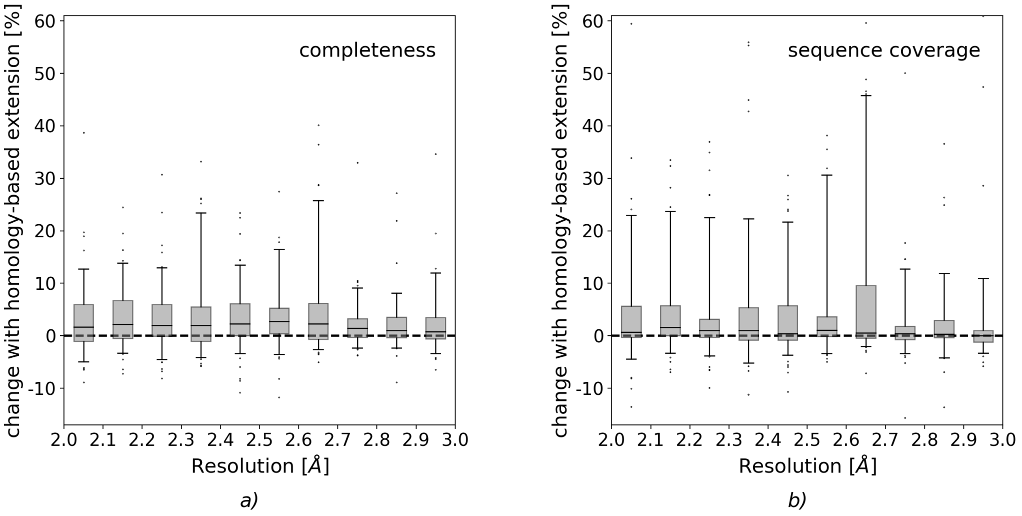

Figure S3 The improvement in model building for test set II at a resolution between 2 and 3 Å as a function of resolution. a) the fraction of residues built; b) sequence coverage. Box plot whiskers correspond to the 5th and 95th percentile.

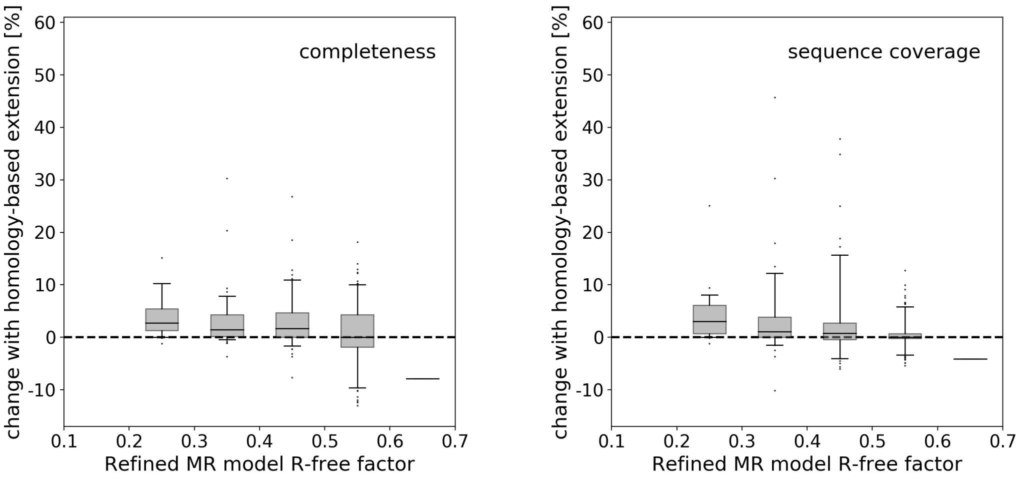

a) *b)*

Figure S4 The improvement in model building for test set II at a resolution between 1 and 2 Å as a function of refined MR model R-free factor. a) the fraction of residues built; b) sequence coverage. Box plot whiskers correspond to the 5th and 95th percentile.

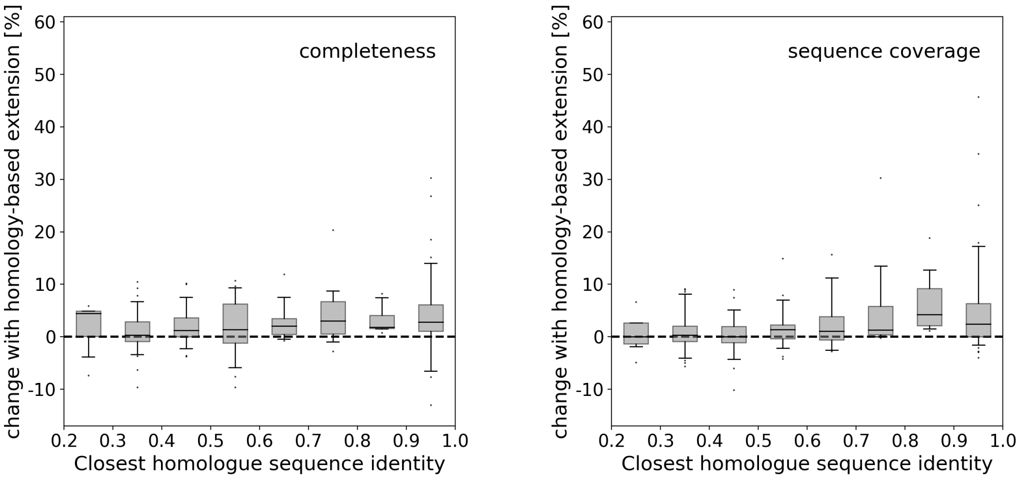

a) *b)*

Figure S5 The improvement in model building for test set II at a resolution between 1 and 2 Å as a function of of sequence identity to a closest homologue used for model extension. a) the fraction of residues built; b) sequence coverage. Box plot whiskers correspond to the 5th and 95th percentile.

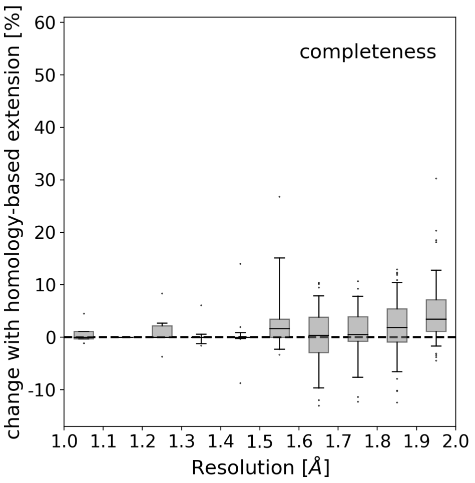

a)

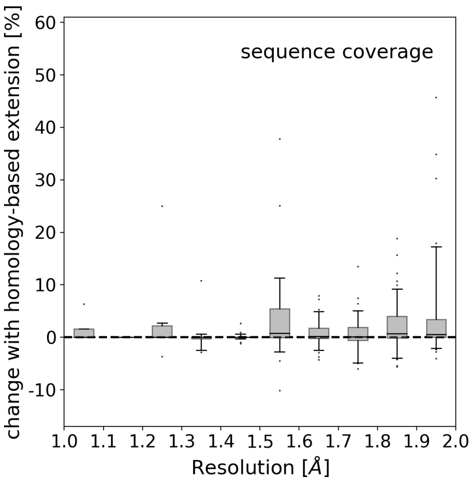

b)

Figure S6 The improvement in model building for test set II at a resolution between 1 and 2 Å as a function of resolution. a) the fraction of residues built; b) sequence coverage. Box plot whiskers correspond to the 5th and 95th percentile.

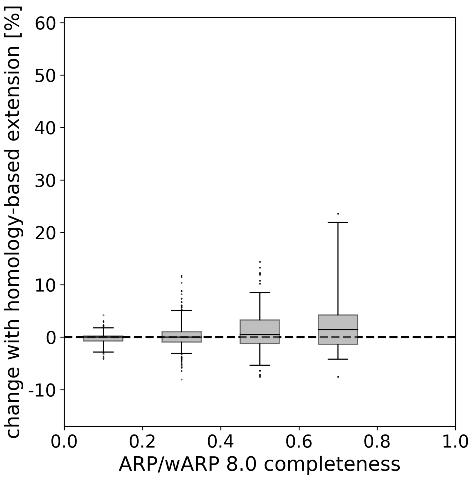

a)

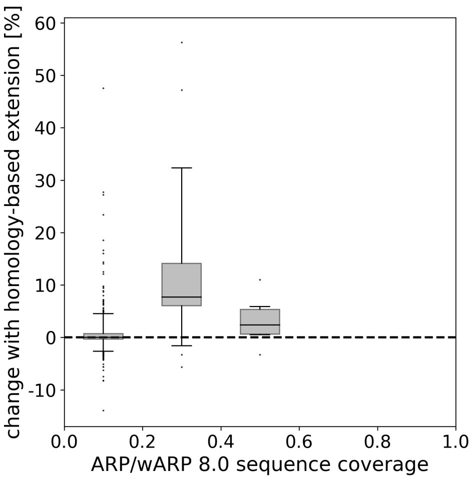

b)

Figure S7 The improvement in model building for test set II at a resolution between 3 and 4 Å as a function of a) model completeness and b) sequence coverage. Box plot whiskers correspond to the 5th and 95th percentile.

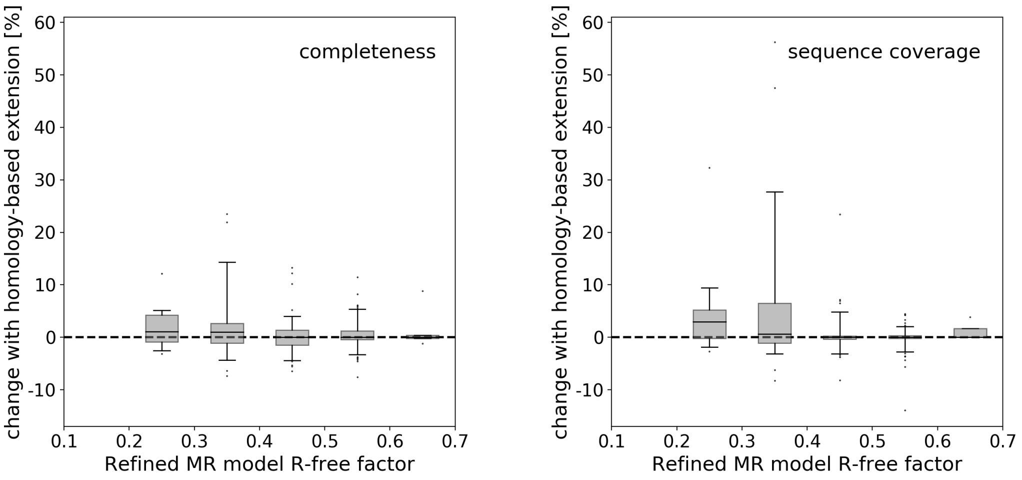

a) *b)*

Figure S8 The improvement in model building for test set II at a resolution between 3 and 4 Å as a function of refined MR model R-free factor. a) the fraction of residues built; b) sequence coverage. Box plot whiskers correspond to the 5th and 95th percentile.

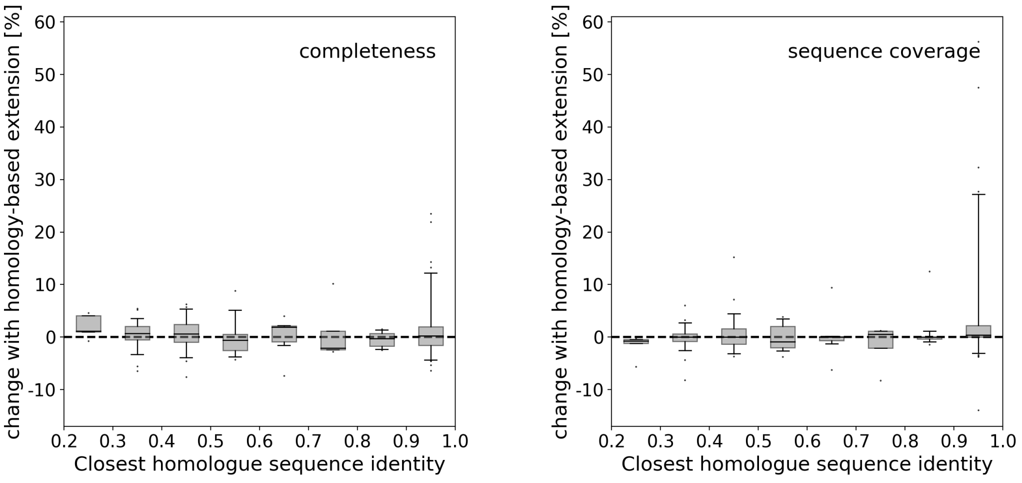

a) *b)*

Figure S9 The improvement in model building for test set II at a resolution between 3 and 4 Å as a function of of sequence identity to a closest homologue used for model extension. a) the fraction of residues built; b) sequence coverage. Box plot whiskers correspond to the 5th and 95th percentile.

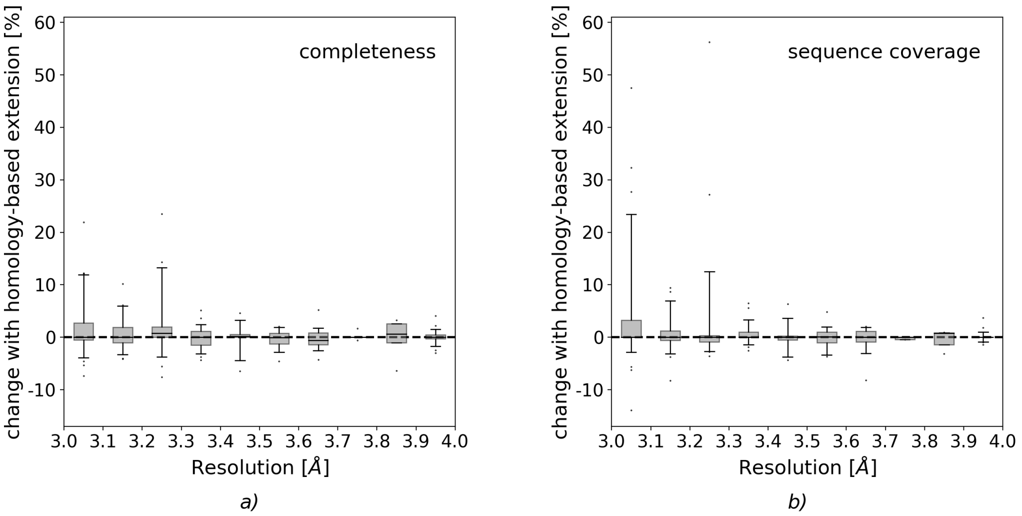

Figure S10 The improvement in model building for test set II at a resolution between 3 and 4 Å as a function of resolution. a) the fraction of residues built; b) sequence coverage. Box plot whiskers correspond to the 5th and 95th percentile.
